# Use of a Porous Alumina Antibiotic-Loaded Ceramic to Treat Bone Defect and Bone Infection After Road Trauma

**DOI:** 10.5435/JAAOSGlobal-D-21-00257

**Published:** 2022-06-21

**Authors:** Deluzarches Philippe, Poli Evelyne, Barrière Guislaine, Denes Eric

**Affiliations:** From the Orthopedic Department, Henri Mondor Hospital, Aurillac, France (Deluzarches); the Research & Development Department, I.Ceram, Limoges, France (Poli, and Barriere); and the Infectious Diseases Department, ELSAN Polyclinique de Limoges, Limoges, France (Denes).

## Abstract

To describe the use of a porous alumina ceramic loaded with antibiotics for the reconstruction of bilateral tibial fractures in a patient who presented with bone loss and infection after a motorcycle road injury.

A 70-year-old man presented open fractures of his both tibiae (proximal involvement on the right side and diaphyseal on the left side). After initial treatment with multiple débridements and the placement of bilateral external fixators, he had bone loss to both tibiae and had developed infections of both legs with multiple organisms identified (*Stenotrophomonas maltophilia*, *Enterobacter cloacae*, and *Pseudomonas aeruginosa*).

We used a porous alumina ceramic, designed according to the defects to fill. This ceramic was loaded with antibiotics (gentamicin and vancomycin). The goal was to obtain locally high concentrations of antibiotics to eradicate bacteria that could have remain in the surgical wound.

Ceramic parts were placed 4 months after the trauma. Local antibiotic concentrations largely exceeded the pharmacological parameters for antibiotics efficacy. External fixators were removed 3 months after implantation. After a follow-up of more than 1 year, there is no relapse of infection, and the patient resumed walking while ceramic parts were left in place and that bone started colonizing ceramic parts.

This ceramic that combines strength and the possibility of antibiotic loading allows thinking of new ways to treat infected fractures with bone loss. Indeed, its mechanical strength provides primary stability, and antibiotics make it possible to secure implantation in an infected area.

Open fractures may lead to bony infections after comminuted, complex fractures. Reconstruction can be challenging because bone graft cannot be used if infection develops in the presence of bone loss. Occasionally, despite proper management, if infection continues, the only option may be an amputation.

One main issue in the management of such situation is the poor diffusion of antibiotics in bone and the bony sequestrum and its inability to sterilize biofilm formed on bone and/or on foreign material. One of the consequences is the need to use high doses of antibiotics, however without the certainty of a cure.

The other issue is the reconstruction after bone loss. Options for reconstruction may rely on medical devices such as metal ones, eventually tailor made, but this can only be performed if there is no infection because of the high risk of contamination of the device.

We report here the use of a porous alumina ceramic to replace bone loss and its loading with antibiotics to protect it from infection in a patient who presented with open fractures and bone loss of both tibiae.

## Case Presentation

**Figure 1:**
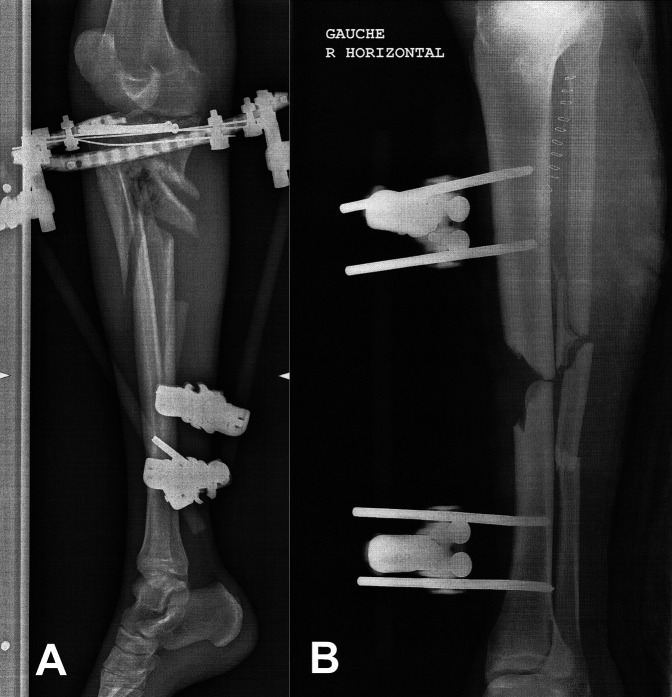
Radiograph after the first surgery for the management in emergency of the road accident: **A**, right side and (**B**) left side

A 70-year-old man presented open fractures and bone loss of both tibiae after a motorcycle accident. On the right side, there was a comminuted fracture at the proximal metaphyseal level (Figure 1, A). He developed a fistula and a *Stenotrophomonas maltophilia* infection. On the left side, there was a diaphyseal bone defect (Figure 1, B) with an *Enterobacter cloacae* infection. This followed initial débridements and the placement of bilateral external fixators and antibiotic-loaded cement to fill the defects. The option recommended by several surgeons, given the bone condition and patient medical history, was bilateral amputation. Comorbidities included diabetes, hypertension, and severe peripheral arterial disease with previous bypasses and stents and the need for a left femoral-popliteal bypass after the initial trauma. Moreover, after the trauma, there was a need for a distal left femoral-popliteal vein bypass. Because we did not have a conventional option to use to allow consolidation of these fractures in the context of infection and we had heard of a loaded ceramic used in bone infections, we turned to this option.

The ceramic used is a porous alumina ceramic (I.Ceram, Limoges, France), which is loaded with antibiotics (vancomycin and gentamicin). The goals are to replace missing bone and to deliver locally high doses of antibiotic to eradicate remaining bacteria in the wound and to protect the device from infection. This antibiotic-loaded device has already been used in orthopaedic surgery for osteomyelitis^1^ and in thoracic surgery to treat deep sternal wound infection with sternum destruction.^2^ Its main characteristics are its high resistance in compression, its osseointegration thanks to its porous, nonabsorbable, and inert structure,^3^ and its capability to deliver very high local doses of antibiotics without toxicity at the site of implantation.^4^

**Figure 2:**
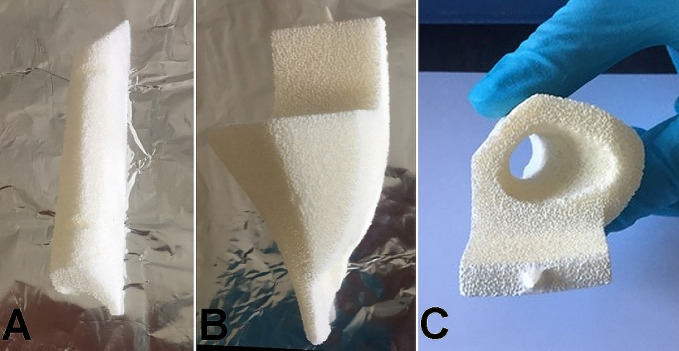
Ceramic parts: (**A**) the one for the left side, lateral view. The one for the right side (**B**) lateral view and (**C**) upper view

**Figure 3:**
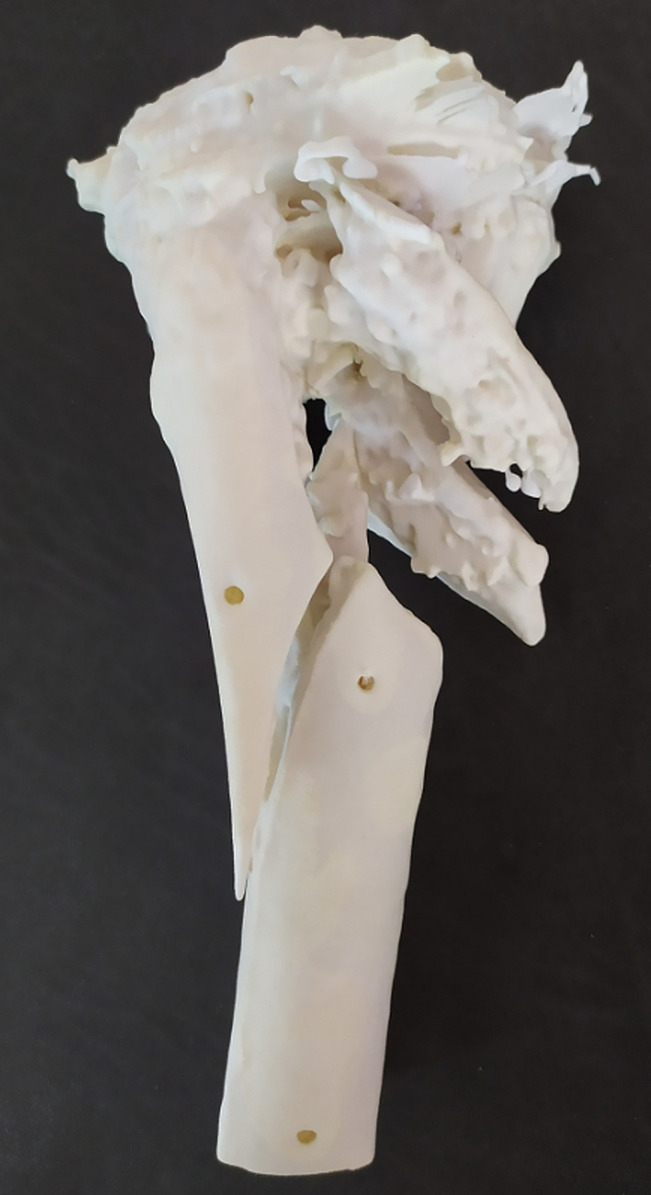
3D plastic printed part made according to the scanner of the right tibia

Shapes of the ceramic (Figure 2, A–C) were tailor made according to the defects. The design of the shapes was aided by 3D modeling and plastic 3D printing of the bones to better visualize the defects and how to replace them (Addidream, Limoges, France) (Figure 3). Loaded antibiotics were 250 mg of vancomycin and 160 mg of gentamicin (in each ceramic) to cover the known bacteria but also some others such as *Pseudomonas aeruginosa* (sensitive to gentamicin), which was found on the right side of bacteriological samples performed during ceramic implantation.

**Figure 4:**
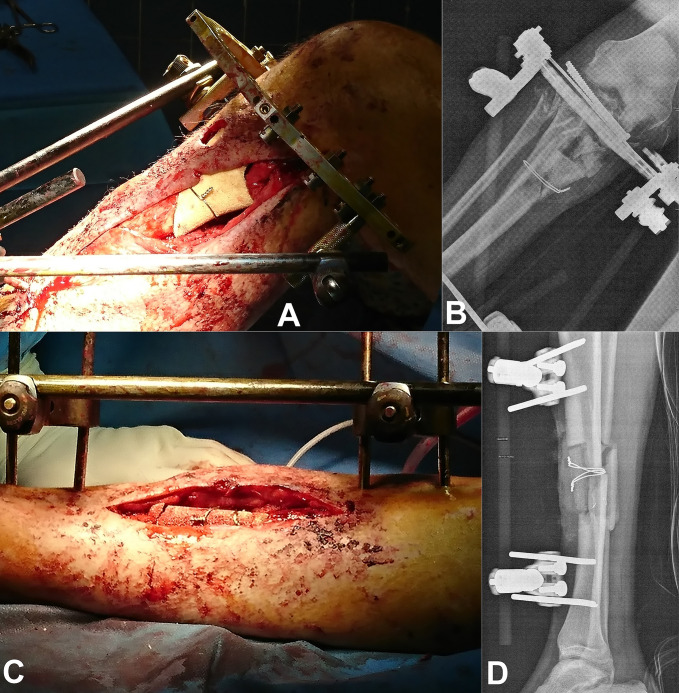
Implantation of the ceramic: **A**, Intraoperative view of the right tibia, (**B**) postoperative radiograph of the right tibia, (**C**) intraoperative view of the left tibia, and (**D**) postoperative radiograph of the left tibia

**Figure 5:**
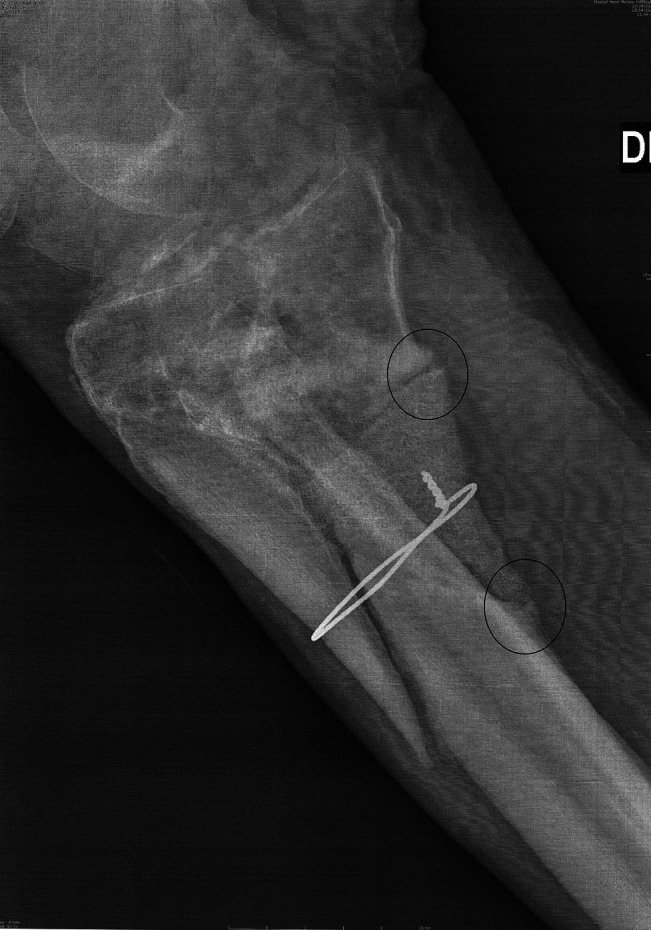
Radiograph of the right tibia performed 7 months after ceramic implantation and 4 months after external fixator removal showing a growth of bone in contact with the ceramic (circles)

**Figure 6:**
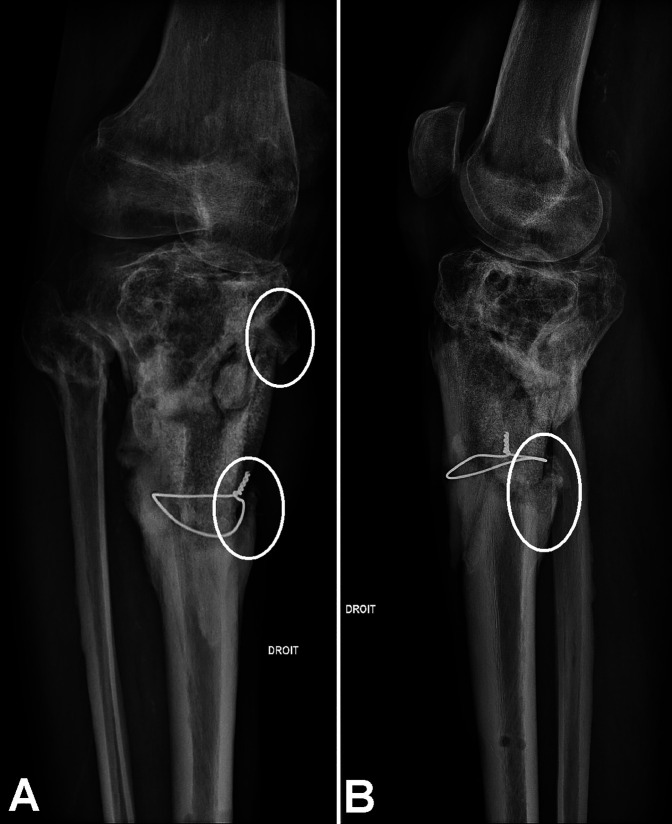
**A**, Front and (**B**) side radiograph of the right tibia performed 18 months after ceramic implantation. White circles indicate bone growth.

For ceramic implantation, cutting guides helped the placement of the devices. This surgery was performed 4 months after the accident. After débridement and cleaning, ceramics were placed and secured with metal straps (Figure 4, A–D). External fixators were left in place during healing for 3 months and then removed. A centromedullary nail was placed in the left tibia to reduce an axial shift because of the use of a monoplane external fixator instead of the previous multiplane one. All bacteriological samples performed during this surgery were sterile. Six months after the accident and 3 months after the use of this ceramic, the patient was allowed to weightbear as tolerated. The ceramic pieces were left in place. Radiograph 6 months after implantation revealed signs of bone growth in contact with the ceramic (Figure 5). After a follow-up of more than 18 months, there is no relapse of infection or fistulae. Radiograph at this time displayed images of ceramic included in bone with bone beginning to encompass the ceramic (Figure 6).

Local dosages, obtained through the Redon drains, of gentamicin after ceramic implantation (peak at H + 3 after ceramic implantation: 951 μmol/L) were greater than 500 times the minimal inhibitory concentration for all bacteria during the first 24 hours (local concentration at H + 24: 352 μmol/L). Of note, the pharmacological parameter testifying the efficacy of aminoglycosides is the maximum concentration/minimal inhibitory concentration that needs to be greater than 10.^5^ This was largely the case for these implantations and that was already observed for the previous patients who benefit from this technology.^2,4^ Vancomycin local concentrations were also very high (peak at H + 3 after implantation: 770 μmol/L with a great Area Under the Curve for 24 hours) allowing to protect the device from gram-positive bacteria colonization. During the same period, no gentamicin (blood concentration < 0.4 mg/L over the 48 hours after implantation) or vancomycin (blood concentration < 4 mg/L during the same period) was detected in blood samples. This local administration reduces the risk of systemic toxicity, which has already been reported.^1,2^ Systemic antibiotic treatment was also prescribed for 3 months targeting bacteria found in samples.

## Discussion

This case highlights the possibilities of new technologies to help surgeons and patients for the management of complex infected bone fractures. This ceramic combines two main characteristics: high resistance to compression offering primary stability after its implantation and its antibiotic loading capability that confers a protection against infections. To our knowledge, no other technology combines these two characteristics. Metal devices have strength but cannot be loaded with antibiotics. Pastes such as Cerament are osteoinductive ceramic and can be loaded with antibiotic but have no mechanical resistance and need an osteosynthesis. Cement has strength and can be loaded with antibiotics, but released amounts are low and cannot guarantee the absence of colonization.^6^

In this case, this antibiotic-loaded alumina ceramic allowed reconstruction, infection protection, and avoid bilateral amputation. This case highlights the possibilities of this antibiotic-loaded ceramic and makes it possible to consider complex surgeries for which the technical possibilities are currently limited.
